# Radiological hazard assessments of radionuclides in building materials, soils and sands from the Gaza Strip and the north of Sinai Peninsula

**DOI:** 10.1038/s41598-021-02559-7

**Published:** 2021-12-01

**Authors:** Mona M. Abd Elkader, Taeko Shinonaga, Mohamed M. Sherif

**Affiliations:** 1 Physics Department, Faculty of Science, Cairo University, Giza, 12613 Egypt; 2 Helmholtz Zentrum München, former Institute of Radiation Protection, 85764 Neuherberg, Germany; 3 Present Address: Institute of Radiation Emergency Medicine, Hirosaki University, Aomori, 036-8564 Japan

**Keywords:** Environmental sciences, Risk factors

## Abstract

Radiological hazards to the residents of the Gaza Strip, Palestine and the north of the Sinai Peninsula, Egypt, were determined using the naturally occurring radionuclides (^226^Ra, ^232^Th and ^40^K) in 69 samples of building materials (demolition debris, plasters, concretes, from recycling plants and raw cements from suppliers), soils and sands collected in the field. The radiological hazard indices and dose rates calculated with the activity concentrations of radionuclides in those materials determined by gamma-ray spectrometry indicate that the values are all within the global limits recommended by the United Nations Scientific Committee on the Effects of Atomic Radiation 2000 and European Commission 1999. The results of Spearman's correlation and hierarchical cluster analysis for ^210^Pb in the building materials, soils and sands suggest that the samples include ^210^Pb from the atmospheric fallout. The medium correlation between ^232^Th and ^40^K in demolition debris implies that their activity concentrations are characteristic of the building materials and constituents of the demolition debris. Non-natural ratio of ^238^U/^235^U was found in the soil and sand samples collected in the Gaza Strip. Furthermore, ^137^Cs and ^241^Am were detected in some soil, sand and demolition debris samples analyzed in this study. The origins of those anthropogenic radionuclides were considered.

## Introduction

All living organisms are continually exposed to ionizing radiation, which has always existed naturally^1^. The sources of that exposure are cosmic rays that come from outer space and from the surface of the Sun, terrestrial radionuclides that occur in the Earth’s crust, in building materials and in air, water and foods and in the human body itself^1^. The radiation exposure in dwellings is mainly due to building material gamma-ray emission from primordial radionuclides such as ^40^K and ^238^U, ^232^Th, ^235^U and their decay products. The typical range of annual radiation dose from natural sources in soil and building materials is reported as from 0.3 to 1 mSv, with the worldwide mean value of 0.48 mSv. The value depends on the type of building materials and the geographical location^2^. Accordingly, it is important to assess and understand the radiation dose due to dwellings for human health and the studies on the radiation exposure from naturally occurring radionuclides have been performed for various regions.

For the Egyptian region, radiological hazard assessments from various building materials were implemented in a previous study. The results showed that radiological hazards of the materials were below the safety limit, expect the granite which showed extremely high activity concentrations^3^. In another study, the radiation levels around Sharm El-Sheikh in South Sinai were estimated. The results indicate that the absorbed dose rates and gamma radiation hazard indices in all locations were higher than the world mean value and unity, respectively^4^.

In the Arabic regions, the activity concentration of ^238^U, ^232^Th and ^40^K in soil samples collected in populated areas of Jordan were reported to be of normal level except for the Zarqa area where significant high ^238^U concentration was found in soil samples originating from phosphate rocks and the values exceed recommended safety levels^5^. In another study, a model of a typical room in Jordanian houses was simulated to calculate the annual effective dose rate for various parts of the room using the Monte Carlo Simulation Program. The results show that the total annual effective dose for Jordanian house was 0.50 mSv year^−1^ with ^238^U contributing about 44% of the total annual effective dose equivalent (AEDE)^6^.

The study on radiological hazard assessment of naturally occurring radionuclides was carried out analyzing ^226^Ra, ^232^Th and ^40^K in the soil and building material samples collected in two cities in Saudi Arabia. The radiological hazard indices were less than the recommended safety limits except for five ceramic samples^7,8^. In the soil samples collected in northern Al Jubail ^137^Cs, ranging from 0.107 to 0.916 Bq kg^−1^, was also detected^7^. Likewise, a radiological map of the whole Kuwait country was established showing the activity concentrations of naturally occurring radionuclides lower than the mean value of worldwide^9^.

The activity concentrations of naturally occurring radionuclides were determined for building materials collected in Taiz and Hodeida, Yemen. They found high activity concentrations of ^226^Ra and ^232^Th in granite and cement bricks. The highest activity concentrations of ^226^Ra and ^232^Th in granites were 154.22 ± 6.97 Bq kg^−1^ and 229.14 ± 3.40 Bq kg^−1^ and in cement bricks were 180.95 ± 6.92 Bq kg^−1^ and 252.85 ± 3.94 Bq kg^−1^, respectively^10^.

In another study, the radiological hazard indices were assessed for 20 soil samples collected at different depths in Hadhramout, Yemen. The mean value of the absorbed dose rate was estimated as 463.12 nGy h^−1^, ranging from 17.22 to 600.16 nGy h^−1^. This value is about eight times higher than the mean global value^11^.

Although there are intensive studies on the assessment of radiological hazards due to residents in various regions, the study relating to the Gaza Strip as well as the north of the Sinai Peninsula is still rare. One of the scarce studies on the Gaza Strip was carried out using solid state nuclear track detector CR-39 to monitor the radon activity concentration and the spatial distribution of radon in beach sand samples of the Gaza Strip. They found a positive correlation between the decrease of grain size of the sediments and radon concentration. The analytical results of radium concentration of the beach sand varied from 0.56 to 8.46 Bq kg^−1^ with the mean value of 1.66 Bq kg^−1^, elevating the activity concentration toward the northern part of the Gaza Strip^12^. For the north of the Sinai Peninsula, the studies on characteristics, spatial distribution and vertical profile of gamma-ray emitting radionuclides in the local harbor, Al-Arish valley and Zaranik for 42 locations were reported. The results indicate that the activities of ^232^Th and ^226^Ra series in samples from the harbor and along the beach were higher than those from non-coastal sites, although no risk exists for public health based on the calculated effective dose equivalent and the recommended limit of 5 mSv year^−1^, except for two locations in the Zaranik^13^.

The past and ongoing siege of the Gaza Strip has led to a severe shortage of building materials since 2007. Many houses, buildings and infrastructure were destroyed and produced huge amounts of demolition debris after the war in the Gaza Strip in 2008. As a result, reuse of the degraded building materials has been required in the past and present. According to the United Nations Development Program (UNDP), the amount of demolition debris in the Gaza Strip, produced from 2008 through 2009, was estimated to be 600,000 tons^14^. The demolition debris was crushed, sieved and reused as recycled building materials. The radioactive substances in those recycled building materials originate mainly from naturally occurring radionuclides in their components such as sandstone, plaster, granite, and others, and anthropogenic radionuclides. Although there are many crushers to recycle demolition debris in the Gaza Strip^15,16^, no information about the radiological hazard from the recycled building materials were reported until now. It is important to understand the radiogenic characters of building materials from the point of view of radiation protection for the residents.

In this study we assessed the radiological hazards from naturally occurring radionuclides (^226^Ra, ^232^Th, and ^40^K) in the building materials, soils and sands collected from the Gaza Strip, Palestine and the north of the Sinai Peninsula, Egypt to discuss the health safety issues due to dwelling in those areas. The study on the recycled building materials is especially important for the area where such materials are required to build houses. The activity concentrations were analyzed by gamma spectrometry and the analytical results were evaluated using various radiological assessment methods. Furthermore, Spearman's correlation and hierarchical cluster analysis were implemented to clarify the correlation among the concentration of radionuclides. Additionally, ^210^Pb, ^235^U, and anthropogenic radionuclides ^137^Cs and ^241^Am were also determined and the results are discussed. The results obtained in this study could form part of database for radiological hazard assessments.

## Material and methods

### Studying area

**Figure 1:**
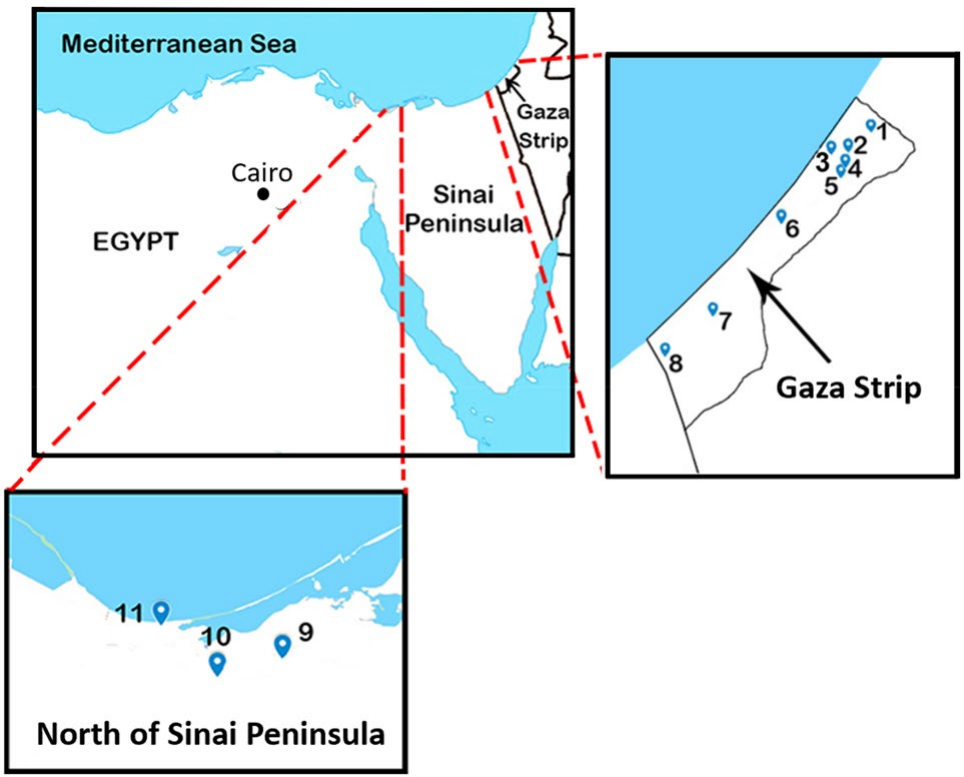
Sampling locations. Gaza Strip: (1) Beit Lahia, (2) Jabalia, (3) El Sheikh Radwan, (4) EL Tuffah, (5) Al Shejaiya, (6) Nuseirat (7) Khan Yunis and (8) Rafah. North of Sinai Peninsula: (9) Negela, (10) Qatia and (11) Rommana.

The present study was carried out with the materials collected in the Gaza Strip, Palestine and the north of the Sinai Peninsula, Egypt, as shown in Fig. 1. The area of the Gaza Strip is 365 km^2^ with around 2.05 million residents^17^. It is located in the southwest of Palestine and on the southeast coast of the Mediterranean Sea. The weather of the Gaza Strip is characterized by a semi-arid climate^18^. The sampling in the Gaza Strip was performed at Beit Lahia, Jabalia, El Sheikh Radwan, El-Tuffah, Al-Shejaiya, Nuseirat, Khan Yunis and Rafah. In the north of the Sinai Peninsula, the samples were collected at Negela, Qatia and Rommana. Rommana located on the coast of the Mediterranean Sea, and Qatia and Negela villages are in a desert plateaus area. The sampling locations are shown in Fig. 1 with blue symbols. 69 samples of building materials, demolition debris, sands and soils were collected: 14 demolition debris, 6 concretes and 5 recycled plasters (KhanYunis, Al Shejaiya and Jabalia), 1 fresh concrete and 2 raw cements (suppliers in the Gaza Strip), 3 raw cements (suppliers in Egypt), 24 soils and sands (18 samples from previous bombed regions and 6 samples from random places in the Gaza Strip) and 14 sand samples (the north of the Sinai Peninsula).

**Figure 2:**
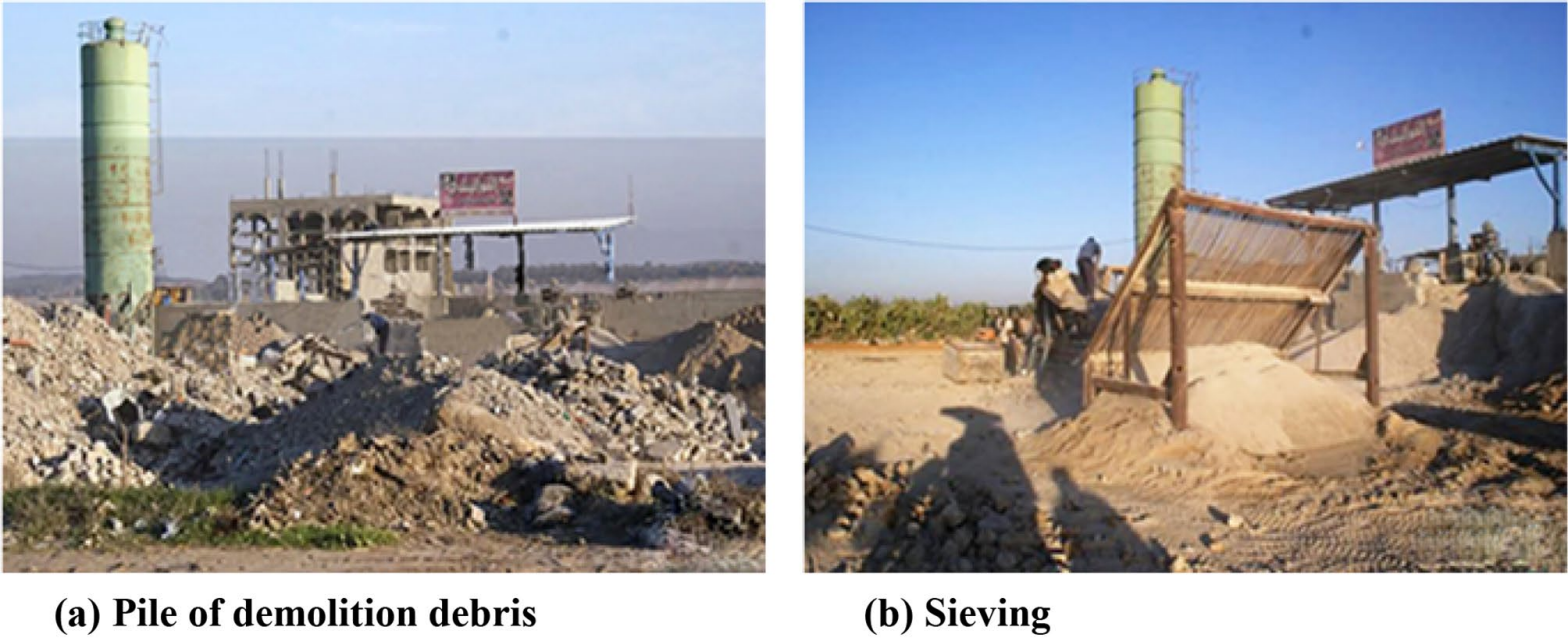
Example of (**a**) pile of demolition debris and (**b**) sieving in the Gaza Strip^19^.

An example of pile of demolition debris and sieving in the Gaza Strip is shown in Fig. 2.

### Sample preparation

The demolition debris and concrete samples were initially broken coarsely using a hammer. Then, the coarse parts were pulverized using a mechanical grinder. The soil samples were packed into polyethylene bags after removing any stones and transferred to the laboratory for analysis. All samples were dried at 85–95 °C until reaching constant weight for each. The dried samples were sieved with a mesh size of 850 µm mesh size, and the samples (< 850 µm) were filled into 113 ml of cylindrical containers having 73.20 mm inner diameter and 26.85 mm height.

The containers were tightly sealed to prevent leakage of gas formic ^222^Rn and ^220^Rn from the samples. The samples were weighed accurately and stored for at least one month prior to gamma-ray measurement for achieving the radioactive secular equilibrium between ^226^Ra, ^228^Ac and their short-lived progenies^20,21^.

### Gamma-ray spectrometry

Activity concentrations of ^226^Ra, ^232^Th, ^40^K, ^238^U, ^235^U, ^210^Pb, ^137^Cs and ^241^Am were determined using high-purity germanium (HPGe) detectors. The gamma-ray spectrometry system consists of HPGe detector, passive shielding, electronic signal processing instrumentation and digital data readout devices. Data acquisition display and analysis of gamma-ray spectra were performed using the Apex-Gamma software.

Energy and efficiency calibrations were performed using the multi-nuclide standard reference source (KA337) and multi-nuclide standard reference solution (QCYA58EK-85013-443), respectively, supplied by Eckert and Ziegler. A diluted solution of the original reference solution in 4 mol L^−1^ HCl was prepared and placed into a plastic case of 73.20 mm inner diameter and 26.85 mm height.

### Self-attenuation and summing coincident correction

The self-attenuation correction factor was experimentally determined through the transmission of gamma-rays emitted from the external point sources (^241^Am, ^152^Eu) through each of the 4 mol L^−1^ HCl mixed standard solution and the sample. The point sources, which cover most of the range of gamma-ray energies, were positioned about 11 cm distant either from the standard or the sample. The measured output gamma-ray intensity (counts s^−1^) was corrected to the measurement date. The self-attenuation correction factor was determined for various energies using the following equation^22^:

1 $$f_{k} = \frac{{In\frac{{T_{sample} }}{{T_{0} }}}}{{\frac{{T_{sample} }}{{T_{0} }} - 1}},$$

where *T*_*sample*_ is the attenuated output gamma-ray intensity (counts s^−1^) from the sample corrected to the measurement date. *T*_*0*_ is the unattenuated output gamma-ray intensity from the reference 4 mol L^−1^ HCl standard solution (counts s^−1^). The final results were calculated using the efficiency for each energy line, the self-attenuation resulting from the differences of the chemical composition and the density between the 4 mol L^−1^ HCL standard and the sample. The radioactivity concentration for a radionuclide in the samples *A*_*si*_ (Bq kg^−1^) was calculated using Eq. (2):

2 $${A}_{si} = \frac{{C}_{ net}}{\gamma\upvarepsilon \left({E}_{\gamma }\right)mt},$$

where *C*_*net*_ is the net counts corrected with self-attenuation, interference and background, *γ* is the emission probability for each certain energy photo peak, *ε*(*E*γ) is the absolute photo-peak efficiency of the germanium detector at this energy, *m* is the mass of the measured sample in kg, *t* is the measurement time (s).

For the most of radionuclides analyzed in this study, the coincidence summing correction was not required. Only for 609.31 keV (^214^Bi) and 583.19 keV (^208^Tl), the sum coincidence correction was required and performed using Genie 2000 software.

### Determination of activity concentration of ^226^Ra, ^232^Th, ^40^K, ^238^U, ^235^U, ^210^Pb, ^137^Cs and ^241^Am

The activity concentration of ^210^Pb, ^137^Cs, ^241^Am and ^40^K was determined using the energy peak at 46.54 keV, 661.66 keV, 59.54 keV and 1460.82 keV respectively^20^.

The activity concentration of ^226^Ra and ^232^Th were calculated based on the weighted mean value of their respective decay products in the secular equilibrium. The activity concentration of ^226^Ra was determined using 259.22 keV and 351.93 keV lines of ^214^Pb, and 609.31 keV and 1764.49 keV lines of ^214^Bi. The activity concentration of ^232^Th was determined using the weighted mean of 968.97 keV and 911.20 keV lines of ^228^Ac, 238.63 keV line of ^212^Pb, 727.33 keV and 1620.74 keV lines of ^212^Bi, and 583.19 keV line of ^208^Tl^20^.

The major gamma lines used for determination of ^238^U were 63.29 keV of ^234^Th [emission probability (ep): 3.73%] and 1001.03 keV of ^234m^Pa (ep: 0.837%)^20,21^. ^234m^Pa can usually be detected by a long time measurement due to the low emission probability of 1001.03 keV gamma line. The gamma line 63.28 keV was used for determination after the correction of self-attenuation and interference by ^232^Th at 63.81 keV (ep: 0.26%). The net counts of ^234^Th were corrected by subtracting the contribution counts of ^232^Th using Eq. (3).

3 $$C_{net} \left( {^{234} Th} \right) = \, C_{total} {-}\left( {\frac{{\left[ {A\left( {^{232}{\text{Th}}} \right)_{weighted \,mean } } \right]\gamma \varepsilon \left( {E_{\gamma } } \right)m t}}{{f_{k} }}} \right),$$

where *C*_*net*_ (^*234*^*Th*) is the net counts of ^234^Th at 63.28 keV peak, *C*_*total*_ is the total counts at 63 keV Peak corrected with the background, *A(*^*232*^*Th)* is the weighted mean activity concentration of ^232^Th calculated from its progeny, γ is the emission probability for 63.18 kev energy line of ^232^Th, *ε*(*E*γ) is absolute photo-peak efficiency of the used germanium detector at this energy and *f*_*k*_ is the self-attenuation correction factor. If the two gamma lines were measured, weighted mean of their activity concentrations was used to determine the activity concentration of ^238^U. Otherwise, the activity concentration of ^238^U was determined using the 63.28 keV line after correction of self-attenuation and interferences.

The major gamma line used for determination of ^235^U in all samples was 185.72 keV, (ep: 57.2%). The line is, however, interfered by ^226^Ra line of 186.21 keV (ep: 3.59%). The gamma line 143.76 keV of ^235^U with low emission probability 10.96%, which is about 20% of the emission probability of 185.72 keV gamma line^20^, was not detected in all measured samples. The correction with a spectral interference by ^223^Ra (t_1/2_ = 11.435d, 144.23 keV, ep: 3.22%) for ^235^U at 143.76 keV peak, if detected, is also required. ^223^Ra was eliminated by subtracting its contributing counts calculated using its 323.87 keV line or the counts of ^219^Rn at 401.81 keV. ^219^Rn is a decay product of ^223^Ra. Since the half-life time of ^219^Rn is very low (t_1/2_ = 3.96 s), it grows rapidly into secular equilibrium with its parent nuclide^23^ and can be used for ^223^Ra activity amount. The interference in the net counts of ^235^U were corrected using Eqs. (4) and/or (5).

4 $$C_{net} \left( {^{235} U \, } \right) \, = \, C_{total} {-}\left( {\frac{{\left[ {A\left( {^{226} Ra} \right)_{weighted \,mean} } \right]\gamma \varepsilon \left( {E_{\gamma } } \right)m t}}{{f_{k} }}} \right),$$

5 $$C_{net} \left( {^{235} U \, } \right) = \, C_{total} {-}\left( {\frac{{\left[ {A\left( {^{223} Ra} \right)_{323.87\, and/ or \,401.81} } \right]\gamma \varepsilon \left( {E_{\gamma } } \right)m t }}{{f_{k} }}} \right){ ,}$$

where *C*_*net*_ *(*^*235*^*U)* is the net counts of ^235^U radionuclide in a peak, *C*_*total*_ is the total counts at this peak, *A(*^*226*^*Ra)*_weighted mean_ is the weighted mean activity concentration of ^226^Ra calculated from its daughters gamma lines, *A(*^*223*^*Ra)* is the activity concentration of ^223^Ra calculated using its gamma line 323.87 keV and/or 401.81 keV of ^219^Rn decay product, $\gamma$ is the emission probability of the corresponding gamma line, *ε*(*E*$\gamma )$ is the absolute photo-peak efficiency of the used germanium detector at this energy and *m* is the mass of measured sample in kg, *t* is the measurement time in seconds and *f*_*k*_ is the self-attenuation correction factor for this energy. If these two gamma lines of ^235^U were detected, weighted mean of their activities was calculated as for the activity concentration of ^235^U. Otherwise, the ^235^U activity concentration was calculated using the 185.72 keV line after correction of background and self-attenuation and interferences.

Background measurements for all energy area were performed regularly, and the results were used in the calculation of final analytical result for each nuclide.

### Calculation of uncertainty

Uncertainty in the activity concentration of radionuclide *i* was calculated using error propagation and Eq. (6):

6 $$\sigma_{i } = (\left( {\sqrt {\left( {\left( {\frac{{U\left( {N_{net} } \right)}}{{N_{net} }}} \right)^{2} + \left( {\frac{U\left( \gamma \right)}{\gamma }} \right)^{2} + \left( {\frac{{U\left( {f_{k} } \right)}}{{f_{k} }}} \right)^{2} } \right)} } \right) \times A_{i} )/m,$$

where *U(*$${N}_{net})$$ = $$\sqrt{{{U}_{t}}^{2}+{{U}_{b}}^{2}+ {{U}_{in}}^{2}}$$ is the uncertainty of the net counts corrected to background counts and the interfering radionuclides, *A*_*i*_ is the activity of radionuclide in Bq, *U*_*t*_*,* *U*_*b*_ and *U*_*in*_ are the uncertainties of the total counts at a certain peak, background counts and interfering radionuclide counts, respectively. *U*($$\gamma )$$ is the uncertainty of the emission probability, *U*($${f}_{k})$$ is the uncertainty of the self-attenuation correction factor, *m* is the mass of the measured sample.

External (*U*_*ext*_) and internal (*U*_*int*_) uncertainties were calculated using Eqs. (7) and (8), respectively, and the larger uncertainty of them is reported in the results.

7 $$U_{ext} = \left( {\sqrt {\left( {\frac{{\sigma_{ex} }}{{\overline{A}_{w} }} } \right)^{2} + (U_{cal} )^{2} } \times \overline{A}_{w} } \right) , \sigma_{ex} = \sqrt {\frac{{\mathop \sum \nolimits_{i}^{N} W_{i } \left( { A_{si} - \overline{A}_{w} } \right)^{2} }}{{\left( {N - 1} \right)\mathop \sum \nolimits_{i}^{n} W_{i} }}} ,$$

8 $$U_{int} = \left( {\sqrt {\left( {\frac{{\sigma_{in} }}{{{\overline{\text{A}}}_{{\text{w}}} }} } \right)^{2} + (U_{cal} )^{2} } \times \overline{A}_{w} } \right), \,\sigma_{{{\text{in}}}} = \sqrt {\frac{1}{{\sum w_{i} }}} ,$$

where ${\overline{A} }_{w}$ is the weighted mean activity concentration (Bq kg^−1^),U_cal_ is the relative uncertainty of efficiency calibration, N is the non-zero weights, A_si_ is the activity concentration of radionuclide i (Bq kg^-1^) contributing to the weighted mean, and wi is the contribution weight of radionuclide i to the weighted mean, equals to $\frac{1}{ {\sigma }_{i}^{2}}$.

### Calculation of radiological hazard indices

The radiological hazard indices were calculated using the activity concentrations of ^226^Ra, ^232^Th and ^40^K obtained in this study expressed in the symbols *A*_*Ra*_, *A*_*Th*_ and *A*_*K*_, respectively, in the following equations.

The gamma index (*I*_*γ*_) is introduced by the European Commission 1999^24^ and is calculated from Eq. (9)^24,25^:

9 $$I_{\gamma} = \left( {A_{Ra} /{3}00} \right) + \left( {A_{Th} /{2}00} \right) + \, \left( {A_{K} /{3}000} \right).$$

The internal hazard index (*H*_*in*_) is calculated using Eq. (10)^26^:

10 $$H_{in} = \left( {A_{Ra} /{185}} \right) \, + \, \left( {A_{Th} /{259}} \right) \, + \, \left( {A_{K} /{481}0} \right).$$

The external hazard index *H*_*ex*_ based on a criterion of Krieger model (1981) was applied and calculated using Eq. (11)^26^:

11 $$H_{ex} = \left( {A_{Ra} /{37}0} \right) \, + \, \left( {A_{Th} /{259}} \right) \, + \, \left( {A_{K} /{481}0} \right)$$

The radium equivalent activity (Bq kg^−1^) is calculated using Eq. (12)^27^:

12 $$Ra_{eq} \left( {{\text{Bq kg}}^{{ - {1}}} } \right) = \, A_{Ra} + {1}.{43}A_{Th} + 0.0{77}A_{K} .$$

The permissible maximum value of the radium equivalent activity is 370 Bq kg^−1^, which corresponds to an effective dose of 1 mSv to the public.

The indoor absorbed dose rate *D*_*indoor*_ (nGy h^−1^) can be calculated using Eq. (13)^24,25^ applying conversion factor of 0.92, 1.1 and 0.08 nGy h^−1^ per Bq kg^−1^ for ^226^Ra (^238^U), ^232^Th and ^40^K, respectively.

13 $$D_{indoor} ({\text{nGy h}}^{{ - {1}}} ) = 0.{92}A_{Ra} + { 1}{\text{.1}}A_{Th} + 0.08A_{K} .$$

The outdoor absorbed dose rate *D*_*outdoor*_ (nGy h^−1^) at 1 meterabove the ground surface is determined using the following equation^28^:

14 $$D_{outdoor} \left( {{\text{nGy h}}^{{ - {1}}} } \right) = 0.{46}3A_{Ra} + \, 0.{6}0{4}A_{Th} + \, 0.0{417}A_{K} ,$$

where the conversion factors used for the calculation of the absorbed dose rate in air (nGy h^−1^) are 0.463, 0.604, 0.0417 for ^238^U, ^232^Th and ^40^K, respectively.

The annual effective dose equivalent (*AEDE*) is calculated using the conversion coefficient from absorbed dose in air to effective dose, 0.7 Sv Gy^−1^, which is found in United Nations Scientific Committee on the Effects of Atomic Radiation (UNSCEAR) 1993^29,30^. For indoor and outdoor measurements, the occupancy factor is approximately 0.8 and 0.2, respectively^29^, as shown in Eqs. (15) and (16).

15 $$AEDE_{indoor} \; (\upmu{\text{Sv}}\,{\text{ year}}^{{ - {1}}} )^{{}} = D_{indoor} \, ({\text{nGy }}\,{\text{h}}^{{ - {1}}} ) \times {876}0{\text{ h}} \times 0.{8} \times 0.{7 } \, ({\text{Sv }}\,{\text{Gy}}^{{ - {1}}} ) \times {1}0^{{ - {3}}} ,$$

16 $$AEDE_{outdoor} \; (\upmu{\text{Sv }}\,{\text{year}}^{{ - {1}}} )^{{}} = D_{outdoor} \, \left( {{\text{nGy }}\,{\text{h}}^{{ - {1}}} } \right) \times {876}0{\text{ h}} \times 0.{2} \times 0.{7 } \, \left( {{\text{Sv }}\,{\text{Gy}}^{{ - {1}}} } \right) \times {1}0^{{ - {3}}} .$$

### Specific absorbed dose rate

The specific absorbed dose rate (*D*_*specific*_) from the materials of pile can be calculated as a source of gamma radiation^25^. The gamma exposure from the materials of pile depends on the facing area (m^2^) and the distance from the pile. In the crushers' case, facing area is assumed to be infinity and the separation distance is 1 m, so the specific absorbed dose rate in air due to the pile of materials is calculated using Eq. (17).

17 $$D_{specific} \left( {{\text{nGy }}\,{\text{h}}^{{ - {1}}} } \right) \, = \, \left( {{47}0A_{Ra} + { 57}0A_{Th} + { 42}A_{K} + { 16}0A_{Cs} } \right) \, \times { 1}0^{{ - {3}}} .$$

Assuming that the workers in crushers work for seven hours per day and six days a week, the annual effective dose (μSv y^-1^) is calculated using Eq. (18).

18 $$AEDE_{specific} \, \left( {\upmu {\text{Sv }}\,{\text{year}}^{{ - {1}}} } \right) \, = D_{specific} \times {2}000{\text{h}} \times 0.{7 }\left( {{\text{Sv }}\,{\text{Gy}}^{{ - {1}}} } \right) \times {1}0^{{ - {3}}} .$$

### Multivariate statistical analysis

The multivariate statistical analysis is used in environmental studies to interpret relations between different variables^31^. Spearman's correlation and cluster analysis were carried out using the IBM SPSS software (version 18.0) to deduce the correlation among the various radionuclides.

## Results and discussion

### Analytical results of certified reference material

To check a reliability of the analytical method used in this study, the standard reference soil, NIST 4353A (Rocky flat soil), provided by the National Institute of standards and technology (NIST) of the US Environmental Measurements Laboratory of the US Departments was analyzed. The reference sample was prepared using the same cylindrical containers for the samples. The analytical results of NIST 4353A were compared to the certified values. The range of the results for all radionuclides analyzed with the 5 detectors used for the sample analysis are corresponding to the 95/95 tolerance limit within the uncertainties as shown in Table 1, indicating a reliability of the analytical method used in this study.

**Table 1:** The range of analytical results for activity concentration of radionuclides in NIST 4353A (Rocky flat soil) obtained by the detectors used for sample analysis. ^a^The analytical results of ^137^Cs were decay corrected to the certified date. ^b^The analytical results of ^241^Am were corrected with ^241^Am ingrowth from ^241^Pu decay and indicated as of the certified date of NIST 4353A. ^c^Not analyzed by alpha spectrometry.

Radionuclide	Certified value (Bq kg^−1^)	95/95 tolerance limit (Bq kg^−1^)	Range of this study (Bq kg^−1^)
^238^U	39.6 ± 3.0	31.9–48.1	33.7–54.4
^235^U	1.88 ± 0.53	0.82–2.68	2.10–2.75
^210^Pb	58.0 ± 9.9	41.8–79.7	47.4–65.3
^137^Cs	21.6 ± 2.6	13.7–30.0	^a^19.3–21.8

	Information value (Bq kg^−1^)	Lower and upper values of the reported results	
^226^Ra	42.4	28.4–52.7	38.7–53.9
^232^Th	73.6	62.1–90.2	73.2–80.8
^40^ K	589	533–719	527–615
^241^Am (gamma spectrometry)	4.7	3.7–6.6	^b^4.29–5.64
^241^Am (alpha spectrometry)	2.5	0.6–5.4	^c^n.a.a

### Activity concentrations of ^226^Ra, ^232^Th, ^40^ K, ^238^U and ^235^U

The analytical results of the activity concentrations of ^226^Ra, ^232^Th and ^40^K, ^238^U and ^235^U for studied samples are presented in Table 2. The activity concentration of ^226^Ra in the building materials is generally higher than that of soils and sands. It was observed that the concentration of ^238^U, ^235^U and ^232^Th in the recycled concretes collected from crushers were two times higher than that of the fresh concrete collected from the local supplier in the Gaza Strip, indicating some concentration of those nuclides during recycling processes. Besides, there is no significant variation of the activity concentration of radionuclides among the demolition debris, recycled plaster and cement within the uncertainties. The radioactivity concentrations of soils collected in the Gaza Strip are nearly two times higher than those in the sands from the same area.

**Table 2:** The mean and range of activity concentrations of ^226^Ra, ^232^Th, ^40^K, ^238^U and ^235^U in the analyzed samples. n: number of analyzed samples. ± : standard deviation. Fresh concrete (Gaza): ± indicates combined uncertainty. *Unable to determine ^238^U due to the low activity concentration of ^238^U and low efficiency of the used detector at low energy area. The activity concentrations are presented using the results of ^226^Ra.

Sample	n	^226^Ra (Bq kg^−1^)		^232^Th (Bq kg^−1^)		^40^K (Bq kg^−1^)		^238^U (Bq kg^−1^)		^235^U (Bq kg^−1^)	
		Mean	Range	Mean	Range	Mean	Range	Mean	Range	Mean	Range
Demolition debris (Gaza)	14	15.6 ± 6.5	5–29	6.2 ± 2.0	1–10	76 ± 25	18–116	16.7 ± 8.2	3–38	0.98 ± 0.45	0.3–1.8
Recycled plasters (Gaza)	5	18.6 ± 5.7	12–25	11.7 ± 5.4	5–20	98 ± 48	41–152	18.8 ± 6.0	12–27	1.25 ± 0.45	0.8–1.9
Recycled concrete (Gaza)	6	20.3 ± 1.8	18–23^34^	7.4 ± 1.6	5–10^34^	60 ± 12	45–77^34^	20.9 ± 2.3	17–24	1.30 ± 0.11	1.1–1.4
Fresh concrete (Gaza)	1	10.49 ± 0.42	–	3.12 ± 0.13	–	43.7 ± 2.8	–	11.6 ± 1.0	–	0.601 ± 0.088	–
Raw cement (Gaza and Egypt)	5	27 ± 12	11–38	13.8 ± 4.5	7–21	65 ± 21	43–100	27 ± 15	11–43	1.83 ± 0.98	0.4–2.7
Soil (Gaza)	9	11.0 ± 4.9	6–23	12.7 ± 6.3	5–25	184 ± 78	110–366	12.6 ± 6.6	5–26	0.97 ± 0.48	0.4–1.9
Sand (Gaza)	15	6.9 ± 2.0	3–12	6.6 ± 1.7	3–11	98 ± 15	72–120	7.0 ± 2.6	3–15	0.54 ± 0.23	0.1–1.2
Sand (Sinai)	14	5.7 ± 1.7	3–11^34^	5.2 ± 1.7	3–10^34^	163 ± 36	117–241^34^	(5.7 ± 1.7)*	(3–11)*	0.29 ± 0.12	0.1–0.6

The activity concentrations of ^226^Ra, ^232^Th and ^40^K in the cement, soil and sand samples from the Gaza Strip and various areas reported previously are shown in Table 3 for comparison. The concentration of ^226^Ra in the cement from the Gaza Strip is within the worldwide range, although that in the soils and sands from the Gaza Strip and the north of the Sinai Peninsula is lower than sample from the other areas by the factor of 3–7, except for Australia, Cyprus and Qatar^27,32,33^. The mean concentration of ^232^Th for the various samples in this study is significantly lower than that of the other areas expect for Cyprus and Qatar. A similar tendency is observed also for ^40^K and its concentration in the cements, soil and sand is clearly lower than that of the world mean and other regions^29^.

**Table 3:** Comparison of activity concentration of ^226^Ra, ^232^Th, ^40^K in cements, soils and sands among various areas in the world.

Sample	Area	^226^Ra (Bq kg^−1^)	^232^Th (Bq kg^−1^)	^40^K (Bq kg^−1^)	References
Cement	Gaza Strip (raw cement)	27 ± 12	13.8 ± 4.5	65 ± 21	This study
	Gaza Strip (plaster)	18.6 ± 5.7	11.7 ± 5.4	98 ± 48	This study
	Australia	52	48	115	^27^
	China	118.7	36.1	444.5	^35^
	Greece	63	24	284	^36^
	Hong Kong	19.2	18.9	127	^37^
	India	37.6	34.3	187. 3	^38^
	Japan	35.8	20.7	163	^39^
	Turkey	40.5	26.1	267.1	^40^
	Slovakia	11.8	18.4	156.5	^41^
	World range	7–180	7–240	24–850	^42^
Soil	Gaza Strip	11.0 ± 4.9	12.7 ± 6.3	184 ± 78	This study
	Cyprus	7.1	5.0	104.6	^32^
	Hong Kong	77.1	146	817	^37^
	India	6	26	501	^38^
	Jordan	42.5	26.7	291.1	^5^
	Pakistan	45	59	648	^43^
	Qatar	17.2	6.4	169	^33^
	Worldwide	32	45	420	^29^
Sand	Gaza Strip	6.9 ± 2.0	6.6 ± 1.7	98 ± 15	This study
	North of Sinai Peninsula	5.7 ± 1.7	5.2 ± 1.7	163 ± 36	This study
	Australia	3.7	40.7	44.4	^27^
	China	32.5	47.7	249.6	^35^
	India	11	130	297	^38^

### Activity concentration of ^210^Pb in samples

The activity concentrations of ^210^Pb in 22 soil and sand samples collected in the Gaza Strip were found to be higher than that of corresponding activity concentration of ^238^U in each sample. The higher activity concentration of ^210^Pb than that of the parent nuclides ^238^U and ^226^Ra imply the presence of unsupported ^210^Pb. Those unsupported ^210^Pb concentrations were calculated using subtraction the ^226^Ra supported activity concentration from the total ^210^Pb activity concentration using the following formula^44,45^: *A*(unsupported ^210^Pb) = *A*(^210^Pb) − *A*(^226^Ra), where *A* is activity concentration of each nuclide. The mean value of activity concentration of unsupported ^210^Pb in the demolition debris, recycled plasters and recycled concrete are 17.80 Bq kg^−1^, 4.67 Bq kg^−1^, 4.08 Bq kg^−1^, respectively, and in soil and sand samples from the Gaza Strip is 4.62, 6.18, Bq kg^−1^, respectively, varying from 0.3 to 28.42 Bq kg^-1^.

**Figure 3:**
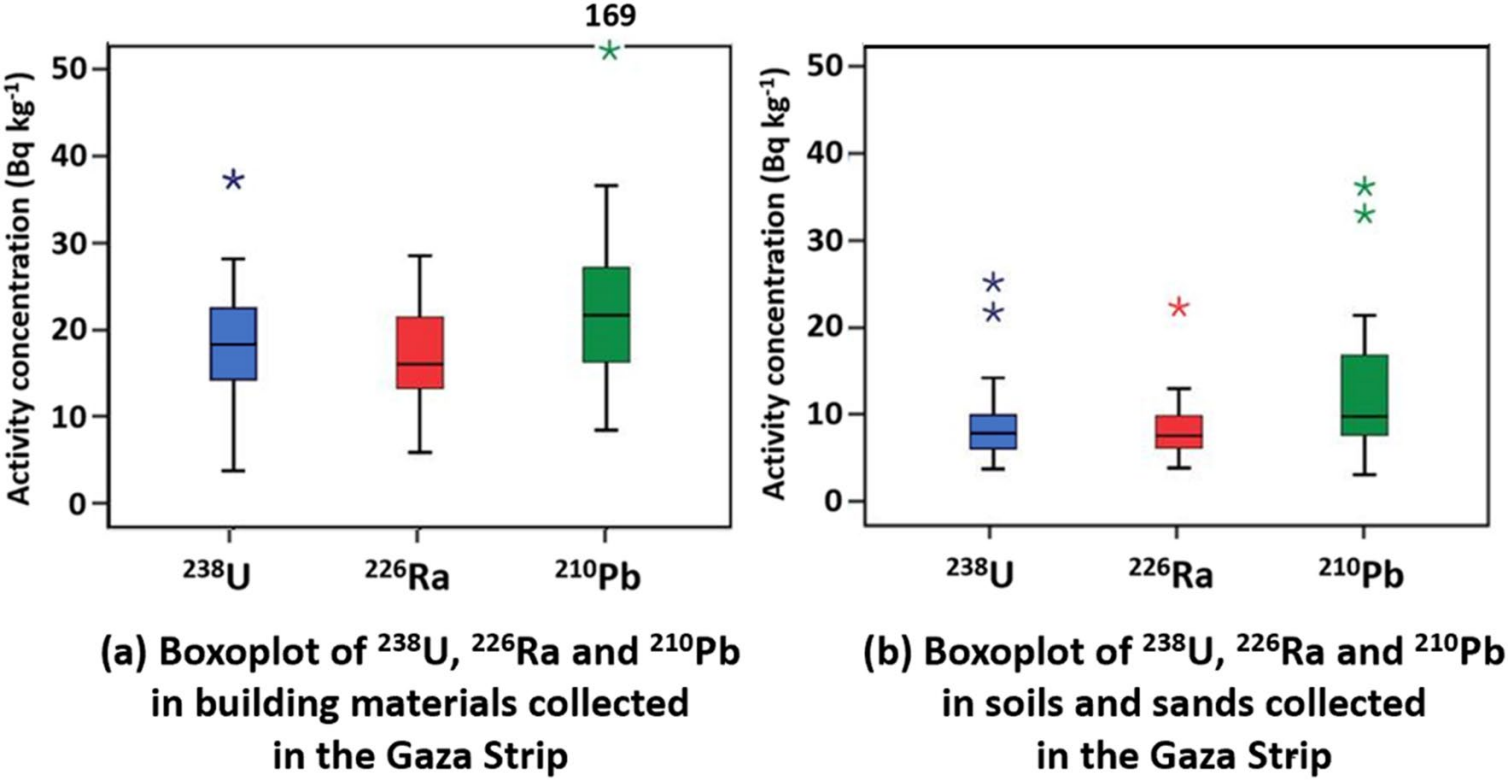
Boxplots of the ^238^U, ^226^Ra and ^210^Pb concentration in the (**a**) building materials and (**b**) soils and sands collected in the Gaza Strip.

Boxplots of the ^238^U, ^226^Ra and ^210^Pb in the building material collected from crushers and soil and sand samples from the Gaza Strip are shown in Fig. 3.

### Activity ratio of ^238^U/^235^U in soil samples from the Gaza Strip

Natural uranium consists of the long-lived radioactive isotopes, mainly, ^234^U, ^235^U and ^238^U, with molar abundances of 0.0057, 0.72, and 99.27%, respectively. In natural uranium, the activity ratio of ^238^U to ^235^U is reported as 21.5^46,47^. In the present study, the activity ratio of ^238^U/^235^U was determined by gamma-ray spectrometry for 24 soil and sand samples collected in the Gaza Strip to understand one of their characters. The activity ratio of ^238^U/^235^U was found as the mean value of 13.2 ± 2.8 and median of 12.7, implying that the soils contain some enriched uranium. In previous study, enriched uranium was observed in crater soil sample collected during the war in the Gaza Strip in 2008. They reported the mean mass ratio of ^238^U/^235^U for three samples based on 12 measurements as 116 ± 16 (natural U: 137.88) in the soils^48^. It can be assumed that the enriched uranium found in this study also contain the spread materials into the atmosphere in the Gaza Strip in relevant events.

### ^137^Cs and ^241^Am in samples

^137^Cs and ^241^Am were detected in some soils, recycled building materials and plaster samples collected in the Gaza Strip and several sand samples from the north of the Sinai Peninsula. The analytical results are shown in Table 4. ^137^Cs are generated by nuclear activities and migrated in the environment. ^137^Cs has the half-life time of 30.17 year and strongly adsorbed to clay minerals, organic materials in the soil, and animal and plant tissues^45,49^. The range of activity concentration of ^137^Cs is 0.06–6.2 Bq kg^−1^ with the highest activity concentration of 6.22 ± 0.22 Bq kg^−1^ observed in a sand sample from Jabal Al Sourani located in the east of El Tuffah, the Gaza Strip. The highest activity concentration in the sand samples from the north of Sinai Peninsula was obtained as 0.27 ± 0.03 Bq kg^−1^ and the value is one order of magnitude lower than that from Jabl Al Sourani. ^137^Cs was detected in 11 demolition debris samples with the highest activity concentration as 2.72 ± 0.10 Bq kg^−1^. The range of concentration for all materials obtained in this study is 0.06–6.2 Bq kg^−1^. In Previous studies, the mean activity concentration of ^137^Cs in top soil samples (0–5 cm depth) collected in Palestine and Lebanon was reported as 7.8 and 23 Bq kg^−1^, respectively^50,51^.

**Table 4:** The mean and range of activity concentrations of ^137^Cs and ^241^Am detected in the samples. n: number of samples in which radionuclide detected; ± : standard deviation. n.a.: not analyzed.

Sample	N	^137^Cs (Bq kg^−1^)		n	^241^Am (Bq kg^−1^)	
		Mean	Range		Mean	Range
Demolition debris (Gaza)	11	0.53 ± 0.79	0.132–2.72	3	0.60 ± 0.17	0.404–0.715
Recycled plasters (Gaza)	2	0.38	0.147–0.614	0	–	< 0.54
Recycled concrete (Gaza)	2	0.146	0.107–0.185	0	–	< 0.57
Soil (Gaza)	7	0.51 ± 0.57	0.064–1.71	3	0.369 ± 0.070	0.298–0.439
Sand (Gaza)	9	1.4 ± 2.0	0.082–6.22	3	0.374 ± 0.085	0.281–0.448
Sand (Sinai)	5	0.203 ± 0.064	0.11–0.27	0	–	n.a

^241^Am has the half-life time of 432.2 y and emits both alpha particles and gamma rays. ^241^Am is a decay product of ^241^Pu (half-life: 14.325 ± 0.006 year)^52^ and its concentration in the environment continues to increase. It was estimated that ^241^Am would reach its maximum activity in the middle of the twenty-first century, supposing no further significant releases of radionuclides would be happened^53^. A quantitative determination of ^241^Am can be carried out by non-destructive analysis using gamma spectrometry, although chemical separation is required for low activity concentrations of ^241^Am^54^. ^241^Am was detected in 3 demolition debris, 6 soil and sand samples from the Gaza Strip. The highest concentration of 0.715 ± 0.054 Bq kg^−1^ for demolition debris sample collected from crushers at KhanYunis. In previous study, the range of ^241^Am concentrations in collected soil samples from Saudi Arabia was reported as 0.021–0.49 Bq kg^−1^^55^ and the values are corresponding to the results obtained in this study. It was reported that ^241^Am was detected in various locations in Algeria as a resultant of radioactive residue from the former French nuclear testing sites in Algeria located about 3200 km from the Gaza Strip^56^. In our previous study, a high Pu concentration in freshly fallen snow was found on Mt. Zugspitze in Germany on the 24th of February 2016, together with a high concentration of PM10 (38 μg m^−3^) compared to the other samples (< 1 μg kg^−1^) from other sampling dates. The modeled backward trajectories on the 23rd of February 2016 indicated that the airborne contribution was transported from the north of the African Continent. Together with the data on activity ratio ^239+240^Pu/^137^Cs, we suggested that the elevated Pu concentration was influenced by the transported Saharan dusts presumably from the source of nuclear tests in Algeria to the European alpine area over a distance of about 2000 km^57^. ^241^Am found in the samples were possibly transported from the former French nuclear testing sites in Algeria or due to other reasons. However, a definitive explanation of the ^241^Am origins was not facilitated in this study.

### Gamma index

The gamma index *I*_*γ*_ is taken into account the dose criteria, distinction of bulk or superficial materials and amounts of the used building materials. For material used in bulk amounts, *I*_γ_ ≤ 1 corresponds to an absorbed gamma dose rate of 1 mSv/y, while *I*_*γ*_ ≤ 0.5 corresponds to an absorbed gamma dose rate of 0.3 mSv year^−1^^24^. The calculated mean of gamma index for various kinds of sample are shown in Table 5. The results indicate that all values are smaller than the maximum limit value of 0.5 corresponding to dose criteria of 0.3 mSv year^−1^.

**Table 5:** Radiological hazard indices calculated for building material, soil and sand samples (mean ± standard deviation), Fresh concrete (Gaza) ± : combined uncertainty, n: number of analysis.

Sample (location)	n	Iγ	H_in_	H_ex_	Ra_eq_ (Bq kg^−1^)	D_indoor_ (nGy h^−1^)	D_outdoor_ (nGy h^−1^)	AEDE_indoor_ (μSv y^−1^)	AEDE_outdoor_ (μSv y^−1^)
Demolition debris (Gaza)	14	0.108 ± 0.016	0.124 ± 0.028	0.082 ± 0.013	30.3 ± 4.7	27.3 ± 4.3	14.1 ± 2.2	134 ± 21	17.3 ± 2.7
Recycled plasters (Gaza)	5	0.153 ± 0.046	0.166 ± 0.049	0.116 ± 0.035	43 ± 13	38 ± 11	19.8 ± 6.0	186 ± 56	24.3 ± 7.3
Recycled concrete (Gaza)	6	0.124 ± 0.015	0.151 ± 0.016	0.096 ± 0.012	35.4 ± 4.3	31.5 ± 3.7	16.3 ± 2.0	155 ± 18	20.0 ± 2.4
Fresh concrete (Gaza)	1	0.0651 ± 0.0018	0.0778 ± 0.0024	0.0495 ± 0.0014	18.32 ± 0.51	16.58 ± 0.47	8.56 ± 0.24	81.3 ± 2.3	10.50 ± 0.30
Raw cement (Gaza and Egypt)	5	0.181 ± 0.044	0.213 ± 0.068	0.140 ± 0.035	52 ± 13	45 ± 12	23.6 ± 6.0	222 ± 57	28.9 ± 7.3
Soil (Gaza)	9	0.162 ± 0.071	0.147 ± 0.064	0.117 ± 0.052	43 ± 19	39 ± 17	20.5 ± 9.0	191 ± 84	25 ± 11
Sand (Gaza)	15	0.089 ± 0.017	0.083 ± 0.017	0.064 ± 0.013	23.9 ± 4.6	21.4 ± 4.0	11.3 ± 2.1	105 ± 20	13.8 ± 2.6
Sand (Sinai)	14	0.099 ± 0.012	0.085 ± 0.013	0.0695 ± 0.0089	25.7 ± 3.3	24.0 ± 2.9	12.6 ± 1.5	118 ± 14	15.5 ± 1.8

### Internal hazard index

Internal hazard index *H*_*in*_ quantifies the internal exposure of ^222^Rn and its decay products. ^222^Rn is a decay product of ^226^Ra, which has a short half-life (3.82 days) and emits alpha particles. The index value *H*_*in*_ should be less than 1 to ensure safe from the exposure of ^222^Rn and their short-lived decay products for the respiratory system of people living in the dwellings. The results of *H*_*in*_ obtained in this study shown in Table 5 are all less than 1.

### External hazard index

External hazard index *H*_*ex*_ is used to evaluate external radiation hazards due to naturally occurring radionuclides. *H*_*ex*_ should be less than 1 to ensure safe exposure radiation levels. For all analyzed samples, *H*_*ex*_ was less than 1 as shown in Table 5, meaning that the external hazards due to the naturally occurring radionuclides are within the safe limit.

### Radium equivalent activity

As the distribution of naturally occurring radionuclides in samples is not uniform, radium equivalent activity (*Ra*_*eq*_) is introduced to represent the activity concentration of ^226^Ra, ^232^Th and ^40^K as a common index, assuming that 370 Bq kg^−1^ of ^226^Ra, 259 Bq kg^−1^ of ^232^Th and 4810 Bq kg^−1^ of ^40^K produce the same gamma-ray dose rate. The permissible maximum value of the radium equivalent activity is 370 Bq kg^−1^, which corresponds to an effective dose of 1 mSv to the public. The calculated values of *Ra*_*eq*_ for various types of sample are shown in Table 5. The mean value of *Ra*_*eq*_ were calculated as 30.3, 43, 35.4, 18.32, 52, 43, 23.9 and 25.7 Bq kg^−1^ for the demolition debris, recycled plasters, recycled concrete, fresh concrete, raw cement, soil (Gaza), sand (Gaza) and sand (Sinai), respectively. All the calculated values of *Ra*_*eq*_ in the samples are found to be less than the permissible maximum value of 370 Bq kg^−1^.

### Indoor absorbed dose rate

To evaluate the absorbed doses in the indoor air (*D*_*indoor*_), the European Commission, 1999 has introduced a scenario for the calculation of the absorbed dose rate in the indoor air in a concrete room with dimensions of 4 m × 5 m × 2.8 m. The thickness and density of walls in this scenario are 20 cm and 2350 kg m^−3^, respectively^24,25^. The results are shown in the seventh column in Table 5. The mean calculated indoor absorbed dose rate due to soil and sand samples collected from the Gaza Strip are 38.8 ± 17.0 and 21.4 ± 4.0 nGy h^−1^. On other hand, the calculated indoor absorbed dose rate due to the recycled concrete ranges from 28.1 ± 0.9 to 37.3 ± 0.8 nGy h^−1^. It is observed that the calculated mean indoor absorbed dose rate in the specified room using recycled concrete is nearly twice times higher than that of fresh concrete.

### Outdoor absorbed dose rate

To assess the exposure to radiation attributed by naturally occurring radionuclides in soil, the outdoor absorbed dose rate (*D*_*outdoor*_) at 1 m above the ground surface was determined. The results are shown in Table 5. The mean of absorbed dose rate of the world is 58 nGy h^−1^^29^. The values obtained in this study are all below the mean of the world.

### Annual effective dose equivalent

The annual effective dose equivalent (*AEDE*) indicates the effective dose received by an adult annually due to the activity concentration of radionuclides present in the building materials. The annual effective dose equivalent for various kinds of samples obtained in this study is shown in Table 5. The mean annual effective dose corresponding to the indoor absorbed dose rate varies from 105 ± 20 μSv year^−1^ (sands from the Gaza Strip) to 191 ± 84 μSv year^−1^ (soils from the Gaza Strip), with the highest value 376 ± 10 μSv year^−1^ for the sample collected from Rafah region in the Gaza Strip. The mean annual effective dose for sand samples collected from the north of Sinai Peninsula is 118 ± 14 μSv year^−1^. According to UNSCER 2008^29^ the worldwide average indoor annual effective dose is 410 μSv year^−1^ and the obtained values are all under that value.

The annual effective dose equivalent *AEDE*_*indoor*_ varies from 81.3 ± 2.3 μSv year^−1^ (fresh concrete) to 155 ± 18 μSv year^−1^ (recycled concrete), indicating that the mean *AEDE*_*indoor*_ for recycled concrete is about two times higher than that of the fresh concrete. This result is corresponding to the values obtained in our previous study using the simulation model for a resident room using Monte Carlo N-Particle Transport(MCNP)^34^.

The UNSCEAR estimated that the mean worldwide exposure to natural radiation sources is as 2400 μSv year^−1^. Total external terrestrial radiation accounts for 480 μSv year^−1^^29^. The mean annual doses in the Gaza Strip and the north of Sinai Peninsula obtained in this study are below this reference level.

### Specific absorbed dose rate of crushers containing demolition debris

Crushers in the Gaza Strip contain huge amounts of demolition debris to be recycled as concrete blocks. The specific absorbed dose rate in air from material pile and the corresponding *AEDE* due to the materials in the crushers are shown in Table 6. The obtained results for the various materials used in the crushers are considered within safe levels. Inhalation doses from the dust generated by crushing the demolition debris and analysis of various demolition debris after the recent war in the Gaza Strip in 2021 are left to the future study.

**Table 6:** Specific absorbed dose rate and the corresponding AEDE in air of the crushers calculated for each material of pile (mean ± standard deviation), n: number of analysis.

Sample (location)	N	D_specific_ (nGy h^−1^)	Range	AEDE_specific_ (μSv y^−1^)	Range
Demolition debris (Gaza)	11	13.8 ± 2.3	9.1–17.1	22.2 ± 3.7	14.6–27.5
Recycled plasters (Gaza)	2	19.9	16.0–23.9	27.9	22.3–33.5
Recycled concrete (Gaza)	2	17.4	15.5–19.2	24.3	21.8–26.9
Soil (Gaza)	7	19.0 ± 9.7	12.1–39.8	27 ± 14	16.9–55.7
Sand (Gaza)	9	12.7 ± 1.4	11.3–15.9	17.8 ± 2.0	15.8–22.3
Sand (Sinai)	5	12.4 ± 2.2	10.0–15.6	17.4 ± 3.1	14.0–21.9

### Spearman’s correlation

To determine the interrelation among the radionuclides (^238^U, ^226^Ra, ^210^Pb, ^235^U, ^232^Th and ^40^K) in the various kinds of samples studied in this study, the analysis of Spearman's correlation was carried out. The Spearman's correlation coefficients among the radionuclides are listed in Table 7. A significantly positive correlation is observed among ^226^Ra, ^238^U, ^210^Pb and ^235^U in the building materials and soil samples of the Gaza Strip, implying a common origin of those radionuclides. In the building material samples, ^232^Th shows an insignificant weak correlation with ^226^Ra, ^235^U and ^238^U and this suggest that they are from various sources of building materials. The medium positive correlation was observed between ^40^K and other radionuclides in soil samples collected from the Gaza Strip.

**Table 7:** Spearman's correlation coefficient (coef.) among ^238^U, ^226^Ra, ^210^Pb, ^235^U, ^232^Th and ^40^K in building materials and soil from the Gaza Strip.

		^238^U	^226^Ra	^210^Pb	^235^U	^232^Th	^40^ K	^238^U	^226^Ra	^210^Pb	^235^U	^232^Th	^40^ K
		Building materials from crushers (Gaza)						Soil and sand samples (Gaza)					
^238^U	Spearman's coef	1						1					
^226^Ra	Spearman's coef	0.890**	1					0.840**	1				
^210^Pb	Spearman's coef	0.859**	0.724**	1				0.657**	0.795**	1			
^235^U	Spearman's coef	0.928**	0.892**	0.811**	1			0.892**	0.828**	0.764**	1		
^232^Th	Spearman's coef	0.220	0.236	0.022	0.243	1		0.675**	0.764**	0.563**	0.706**	1	
^40^K	Spearman's coef	− 0.280	− 0.260	− 0.256	− 0.312	0.507*	1	0.579**	0.605**	0.527**	0.702**	0.765**	1

**Correlation is significant at the 0.01 level (2-tailed). *Correlation is significant at the 0.05 level (2-tailed).

### Hierarchical cluster analysis

The interrelations among the radionuclides can be well classified and qualitatively visualized using hierarchical cluster analysis (HCA). In HCA, similar variables/cases are classified in a same group. Clustering is repeated with the next most similar variables till all of them are clustered^31^.

**Figure 4:**
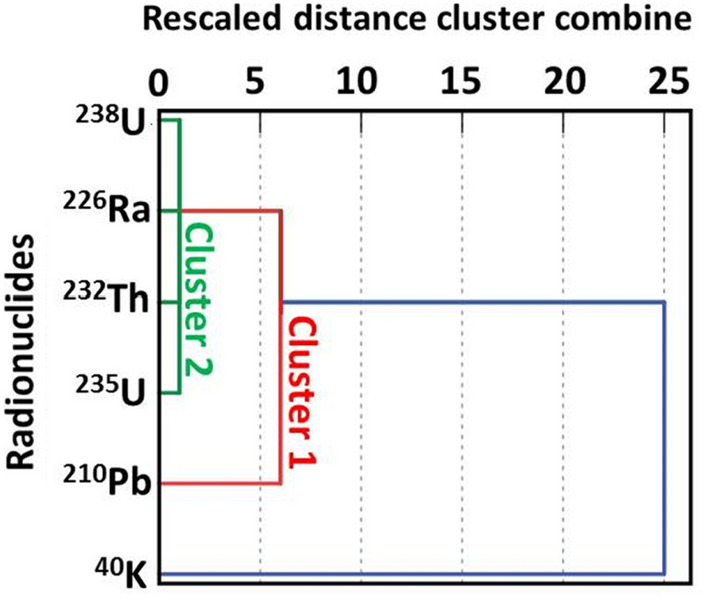
Dendrogram using single linkage. The dendrogram shows HCA among naturally occurring radionuclides in building materials from crushers in the Gaza Strip.

Visual representation of the distance at which clusters are combined can be carried out using dendrogram, reading from left to right. Vertical lines show joined clusters. The position on the scale indicates the distance at which the clusters are joined. The observed distances are rescaled (not actual distance) and it ranges from 1 to 25. The ratio of the rescaled distances within the dendrogram is the same as the ratio of original distances. The dendrogram was agglomerated with the nearest neighbor method using squared Euclidean distance. The derived dendrogram for building materials collected from the Gaza Strip is shown in Fig. 4. The radionuclides in the dendrogram are classified into two clusters. The blue line includes single radionuclide ^40^K, not cluster. Cluster 1 (red line), consisting of ^210^Pb and ^226^Ra, shows little similarity with the other radionuclides, as ^210^Pb derives not only from the decay of ^238^U series but also from atmospheric fallout. Cluster 2 (green line) includes ^238^U, ^226^Ra, ^232^Th and ^235^U, indicative of their main origin in the natural radioactive series of the earth crust.
